# Performance of CuAl-LDH/Gr Nanocomposite-Based Electrochemical Sensor with Regard to Trace Glyphosate Detection in Water

**DOI:** 10.3390/s20154146

**Published:** 2020-07-25

**Authors:** Chuxuan Zhang, Xinqiang Liang, Yuanyuan Lu, Hua Li, Xiangyang Xu

**Affiliations:** 1 College of Environmental and Resource Sciences, Zhejiang University, Hangzhou 310058, China; 21814101@zju.edu.cn (C.Z.); xuxy@zju.edu.cn (X.X.); 2 Key Laboratory of Water Pollution Control and Environmental Security Technology, Hangzhou 310058, China; 3 Physical and Theoretical Chemistry Laboratory, University of Oxford, Oxford OX1 3QZ, UK; yuanyuan.lu@sjc.ox.ac.uk; 4 Institute of Environment, Resource, Soil and Fertilizer, Zhejiang Academy of Agricultural Sciences, Hangzhou 310021, China; lisar2002@zju.edu.cn

**Keywords:** electrochemical detection, glyphosate, layer double hydroxides, sensor

## Abstract

Glyphosate, which has been widely reported to be a toxic pollutant, is often present at trace amounts in the environment. In this study, a novel copper-aluminum metal hydroxide doped graphene nanoprobe (labeled as CuAl–LDH/Gr NC) was first developed to construct a non-enzymatic electrochemical sensor for detection trace glyphosate. The characterization results showed that the synthesized CuAl–LDH had a high-crystallinity flowered structure, abundant metallic bands and an intercalated functional group. After mixed with Gr, the nanocomposites provided a larger surface area and better conductivity. The as-prepared CuAl–LDH/Gr NC dramatically improved the enrichment capability for glyphosate to realize the stripping voltammetry detection. The logarithmic linear detection range of the sensor was found to be 2.96 × 10^−9^–1.18 × 10^−6^ mol L^−1^ with the detection limit of 1 × 10^−9^ mol L^−1^ with excellent repeatability, good stability and anti-interference ability. Further, the sensor achieved satisfactory recovery rates in spiked surface water, ranging from 97.64% to 108.08%, demonstrating great accuracy and practicality.

## 1. Introduction

Glyphosate (common commercial name: “Roundup”), a widely used organophosphorus herbicide, has the advantages of low cost, high efficiency and broad-spectrum [1]. Since the late 1970s, the annual dosage of glyphosate to plants has increased approximately 100-fold, and its residues or metabolites have been found in 45% of the topsoil collected from eleven countries and six crop systems [2,3]. The glyphosate can induce acute poisoning and cause liver, kidney and cardiovascular damage; moreover, severe cases can be life-threatening in humans. Even in trace concentrations, glyphosate has long-term toxicity and adverse chronic effects [4,5]. Besides, it can actively interact with other organic compounds, causing persistent pollution in food and grains [6]. The International Agency for Research on Cancer (IRCA) has classified glyphosate as “probably carcinogenic to humans”, prompting serious safety concerns [7]. To avoid health risks, it is crucial to develop a rapid and convenient glyphosate detection technique.

High-performance liquid chromatography (HPLC) and mass spectrometry (MS) have been employed for glyphosate detection [8,9,10,11]. In spite of the high selectivity and sensitivity, some inherent limitations, such as high cost, complexity of operation and cumbersome pretreatments, make them unsuitable for practical applications. With the development of nanomaterials and electronic devices, electrochemical detection has gradually advanced, especially in terms of the detection of heavy metals [12]. Electrochemical sensors can be more effective for pesticide detection, due to high accuracy and portability [13,14].

Owing to the poor electro activity, it is hard to realize the electrochemical detection of glyphosate without electrode modification [15]. Jafar et al. used simple gold electrodes to directly detect glyphosate with a detection limit up to 2 mol L^−1^ [5]. Therefore, the detection of glyphosate needs electrode modifications to enhance measurable signals. Various enzymes, including urease, horseradish peroxidase and tyrosinase, are used to quantify glyphosate by the inhibition of the catalytic activity [16,17,18]. However, the denaturation and digestion of enzymes greatly limited the performance of such biosensors. Besides, other toxic substances, such as heavy metal ions, or extreme pH conditions may also affect the performance of such sensors by generating a false positive current [19]. To overcome these limitations, non-enzymatic sensors based on metallic modifications have been developed. Minh Huy et al. assembled a molecularly imprinted sensor that uses glyphosate as a template via the electro-polymerization of p-aminothiophenol-functionalized gold nanoparticles on the electrode surface for glyphosate detection, with a linear range of 1.69 × 10^−10^–1.69 × 10^−4^ mol L^−1^ [20]. Khenifia et al. prepared a non-enzymatic sensor via the electrodeposition of a thin nickel layer to detect glyphosate [21]. The sensor employed Ni (III) centers to electro-oxidize the amino group in glyphosate, which had a detection range of 1.0 × 10^−5^–9 × 10^−4^ mol L^−1^. Based on the chelation between Ag+ and glyphosate, Li et al. prepared a photoelectrochemical sensor to detect glyphosate. The linear range of this sensor was 1.0 × 10^−10^–1.0 × 10^−3^ mol L^−1^ [22]. Layered double hydroxides (LDHs) have gained increasing attention as two-dimensional host/guest nanomaterials in several applications, including catalysis, biochemical applications, fire retardants, adsorption, super capacitors and electrochemical sensors [23,24,25,26,27,28]. Divalent and trivalent metal ions with a radius close to that of Mg^2+^ (e.g., Al, Ni, Mn, Co and Cu) can change the metal composition in LDHs [29]. The open structure and high biocompatibility of LDHs prompt their extensive use as effective redox mediators or stable matrixes in diverse electrochemical sensors, including the NiMn-LDH/graphene oxide for monitoring sugars and peroxides, the NiAl-LDH-based sensor for detecting ozone, and the Co_2_Al-LDH/graphene-based on a biosensor to measure trichloroacetic acid [30,31,32]. In these cases, the LDHs exhibit extraordinary catalytic properties and a well-defined redox peak.

Compared with other metals, copper has the advantage of high electrochemical activity, low resistance and excellent catalytic performance. Besides, Cu-based nanomaterials have showed a high affinity for glyphosate in a previous study [33]. To our knowledge, LDH-based sensors have primarily employed traditional Mg- or Ni-centered LDHs and only one paper about the CuAl-LDH sensor for glucose detection using amperometry has been reported [34]. In previous studies, hydroxides (LDHs) as a host can construct a host–guest structure, which could effectively, selectively insert pesticides or phenolic compounds into the interlayer space [35]. However, there has been a paucity of studies on the application of Cu-based LDHs, especially in pesticide detection. Furthermore, the integration of Cu nanocomposite with carbon nanomaterials like graphene can boost its catalytic activity [36].

Here, we explored the performance of a CuAl-LDH/graphene nanocomposite (denoted as CuAl-LDH/Gr NC) based electrochemical sensor for trace glyphosate detection. The CuAl-LDH was prepared via the facile co-precipitation method and directly ultrasonic mixed with graphene. Under optimal conditions, the sensor exhibited a low detection limit for glyphosate, as well as good stability and anti-inference. Finally, we used the sensor in real water samples and obtained the satisfactory recovery rates.

## 2. Experimental

### 2.1. Chemicals and Apparatus

Chitosan, graphene, glyphosate and other organophosphorus standard solutions were purchased from Aladdin (Shanghai, China). Nafion (5%) was purchased from Sigma-Aldrich. Acetic acid (HAc), sodium acetate (NaAc), sodium hydroxide (NaOH), copper nitrate hydrate (Cu(NO_3_)_2_·3H_2_O), aluminum nitrate nonahydrate (Al(NO_3_)_3_·9H_2_O), sodium carbonate (Na_2_CO_3_), potassium ferricyanide (K_4_[Fe(CN)_6_]) and potassium ferrocyanide (K_2_[Fe(CN)_6_]) were purchased from Sinopharm Chemical Reagent Co., Ltd. Ultra-pure water was used in all experiments.

The morphology of the CuAl-LDH/Gr NC materials was observed using a scanning electron microscope (SEM, JSM-5600LV JEOL, Japan); energy dispersive spectroscopy (EDS) and an X-ray photoelectron spectroscope (XPS, Thermo Scientific K-Alpha) were employed for the elemental analysis of the synthetic CuAl-LDH. X-ray diffraction (Rigaku Ultima IV, Japan) and Fourier transform infrared spectroscopy (FTIR, Nicolet iS5, USA) were used to analyze the characteristic peaks and crystallite structure of the synthetic CuAl-LDH; a CHI660e electrochemical workstation (Shanghai Chenhua Co., Ltd., Shanghai, China) were used for electrochemical experiments. All electrochemical experiments were conducted using three-electrode systems (glassy carbon electrode (GCE, d = 3 mm), Ag/AgCl electrode and Pt electrode as the working electrode, reference electrode and counter electrode, respectively).

### 2.2. Synthesis of CuAl-LDH Composites

The CuAl-LDH composites were synthesized according to the coprecipitation method with appropriate modifications [34]. Firstly, 0.1 g of chitosan was dissolved in 50 mL of a 1% acetic acid (HAc) solution. Then, 4.53 g (0.018 mol) of Cu(NO_3_)_2_·3H_2_O and 2.34 g (0.006 mol) of Al(NO_3_)_3_·9H_2_O were added to the solution, with the Cu/Al molar ratio of 3:1, under vigorous stirring. Furthermore, 1.33 g (0.0125 mol) of Na_2_CO_3_ was added to 50 mL of water to obtain a carbonate solution. Then, the Cu/Al nitrate solution was added to the carbonate solution under magnetic stirring to maintain the pH within the range of 8.0–8.5. The resultant suspension was stirred vigorously at 30 °C for 60 min to ensure a complete reaction. The blue-black colored CuAl-LDH composites were obtained by centrifuging (3000 rpm, 5 min) the resultant suspension and washing the composites thrice with water and ethanol, alternately. Finally, the obtained composites were dried at 80 °C for 24 h. After cooling to room temperature, the obtained composites, the CuAl-LDH nanocomposites were prepared.

### 2.3. Fabrication of CuAl-LDH/Gr-Modified GCE

Pristine graphene was facilely preprocessed according to a method described in previous papers [37]. Then, the CuAl-LDH nanocomposites and the preprocessed Gr solution were mixed (mass ratio = 10:3) into 10 mL of ethanol solution and dispersed ultrasonically for 30 min to obtain the CuAl-LDH/Gr suspension. Before modification, the GCE was polished using alumina slurry (1.0 and 0.05 μm) and ultrasonically cleaned in ultrapure water and ethanol. The polished electrodes were scanned using cyclic voltammetry (CV) in 0.1 M KCl solution containing 5 mM of [Fe(CN)_6_]^3−/4−^ until the peak-to-peak potential differences were less than 85 mV. Then, 16 μL of the CuAl-LDH/Gr suspension was uniformly dropped onto the surface of the GCE; then, the electrode was dried at room temperature. After drying, 10 μL of a Nafion–ethanol solution (1 wt %) was drop-casted on the nanocomposite to further immobilize the CuAl-LDH/Gr. As controls, a Gr-modified electrode (Gr/GCE) and a CuAl-LDH-modified electrode (CuAl-LDH/GCE) were also prepared using the same method.

### 2.4. Electrochemical Behavior Tests

To explore electron’s transfer reaction mechanism of the different modified electrodes, CV tests were conducted. First, the bare GCE, freshly prepared Gr/GCE, CuAl-LDH/GCE and CuAl-LDH/Gr/GCE were individually immersed into separate 0.1 M KCl solutions containing 2 mM of [Fe(CN)_6_]^3−/4−^ to compare their electrochemical properties. Further, the CV responses of the aforementioned electrodes were recorded in a 0.2 M acetic acid buffer solution (ABS) to verify the probe function of the CuAl-LDH/Gr NCs. Then, the CuAl-LDH/Gr-modified electrode was immersed in 0.2 M ABS solutions with different concentrations of glyphosate to observe the signal changes. Electrochemical impedance spectroscopy (EIS) tests were performed in a 0.1 M KCl solution with 5 mM of [Fe(CN)_6_]^3−/4−^ (1:1), by setting the frequency from 0.01 to 106 Hz under an amplitude potential of 50 mV.

The optimization experiments were carried out in a 0.2 M ABS solution containing 40 ppb glyphosate by differential pulse voltammetry (DPV). The intensity of the electrochemical response was recorded based on the reduction of the initial peak current (ΔI = I_t_ − I_0_; I_t_ and I_0_ represent, respectively, the peak current for the cases with and without glyphosate).

## 3. Results and Discussion

### 3.1. Characterization of CuAl-LDH/Gr Nanocomposites

**Figure 1:**
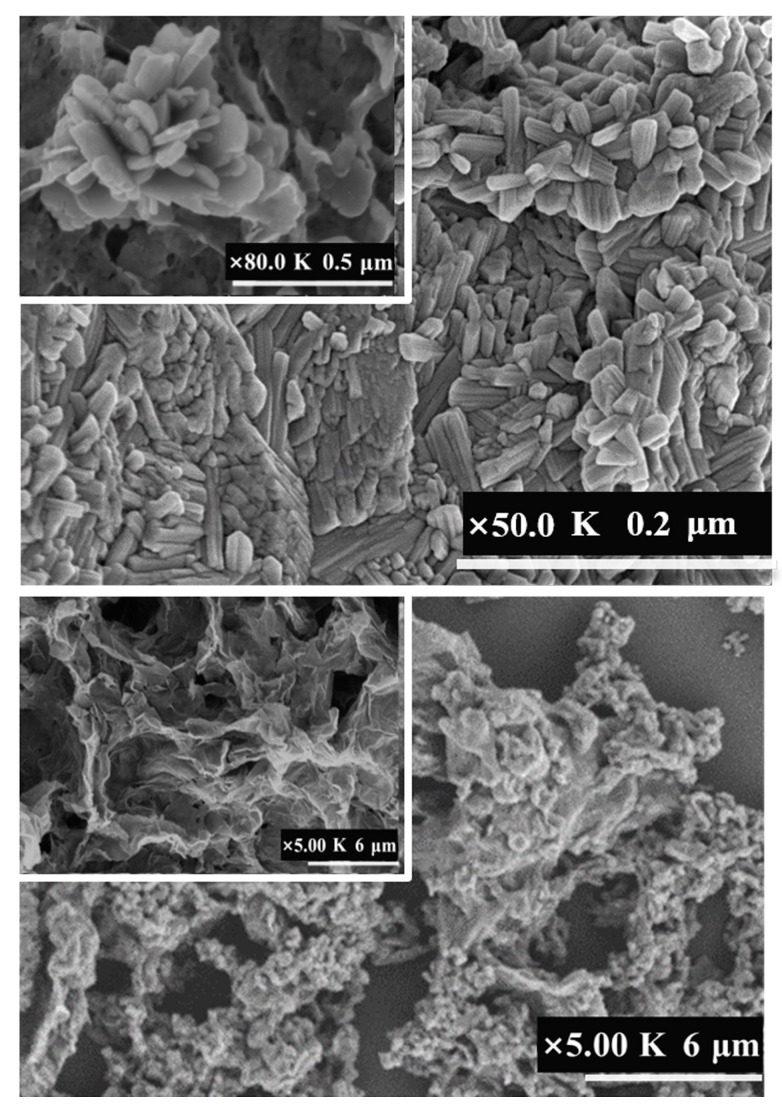
(**a**) Scanning electron microscope (SEM) of CuAl-LDH (×50.0 k; CuAl-LDH inset ×80.0 k) and (**b**) CuAl-LDH/Gr NCs (×5.00 k; Gr inset ×5.00 k).

The SEM image of as-prepared CuAl-LDH was shown in Figure 1a. When Cu(NO_3_)_2_ and Al(NO_3_)_3_ at a molar ratio of 3:1 were mixed in 0.2 wt % chitosan acetic acid solution, the complete CuAl-LDH had a chain-like pattern. At 80.0k magnification, the single CuAl-LDH had a flower morphology, whose diameter was 0.5 μm and petals were serrated. In Figure 1b, a large number of stacked CuAl-LDH was dispersed on the lined surface of graphene forming a cross-connected three-dimensional (3D) structure. This nanoscale structure of CuAl-LDH/Gr not only provides more binding sites for glyphosate, but also improves the conductivity by a larger electron-exchange surface on the GCE.

**Figure 2:**
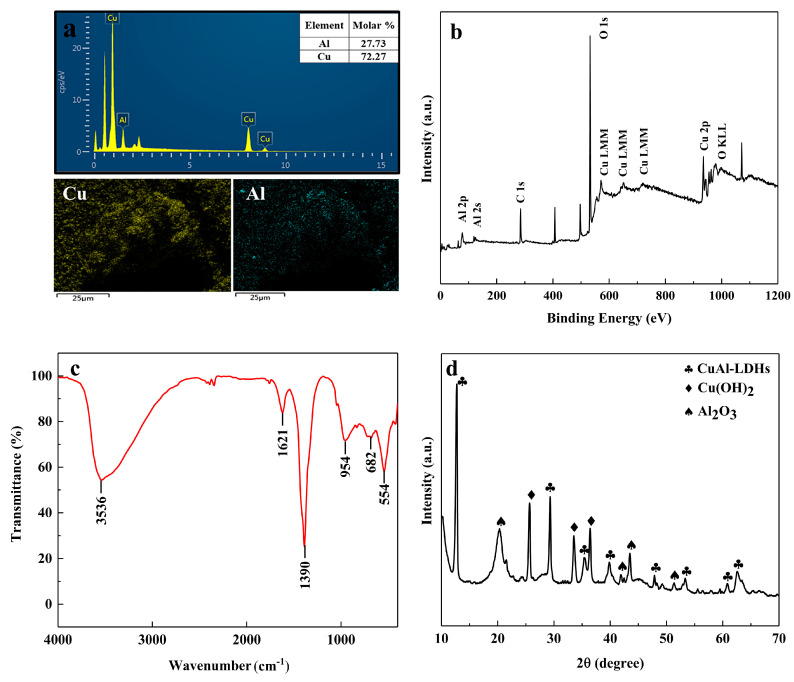
(**a**) Energy dispersive spectroscopy (EDS); (**b**) X-ray photoelectron spectroscopy (XPS) image; (**c**) Fourier transform infrared spectroscopy (FTIR) spectra and (**d**) X-ray powder diffraction (XRD) pattern of CuAl-LDH.

The Cu and Al distributions on the surface of the as-prepared product were evaluated by EDS mapping (Figure 2a), in which Cu and Al were tightly doped, suggesting homogeneous dispersion. The M(II) or M(III) molar ratio x in the range of 0.2–0.33 about the LDH general formula ([M^2+^_1−x_M^3+^_x_ (OH)_2_]^x+^ (A^n−^)_x/n_ mH_2_O) is usually considered the LDHs more suitable for stable structure and composition, so the elemental ratio 27.73% of as-prepared LDH was satisfactory [29]. According to the elemental content ratio and charge balance, the as-prepared product was inferred as [Cu_0.72_Al_0.28_(OH)_2_] [(CO_3_^2−^)0.14·mH_2_O]. The XPS images showed the full range spectrum peaks of the CuAl-LDH (Figure 2b). The photoelectrons split by the spin orbits of Cu 2p_3/2_ and 2p_1/2_ at 933.4 eV and 953.2 eV, respectively confirmed the Cu^2+^ oxidation state [38]. Simultaneously, shake-up peaks of Cu^2+^ were observed at 943.2 eV and 962.5 eV. The peak binding energy in 78.08 eV and 129.08 eV corresponded to Al 2p and Al 2s, respectively [34]. Moreover, the quantitative analysis of XPS was similar to the EDS results and further confirmed the composition of CuAl-LDH.

The FTIR spectra showed the characteristic groups and bands in as-prepared CuAl-LDH (Figure 2c). The absorption bands below 1000 cm^−1^ were typically from metal atomic vibration such as the M-O, M-O-M and O-M-O bonds, thus bands ranged from 544 to 954 cm^−1^ corresponding to Cu-O or Al-O [39]. The strong peak at 3536 cm^−1^ with the 1621 cm^−1^ bending mode that confirmed the presence of the intercalated OH^−^, H_2_O and CO_3_^2−^ was identified by the strong absorption peak at 1390 cm^−1^ [39]. These intercalated structures and abundant metallic bands in CuAl-LDH will provide more combination sites for the interaction and immobilization of glyphosate. The reflection of as-prepared product in the XRD pattern at 2θ of 11.2°, 28.2°, 35.1°, 39.8°, 47.3°, 60.0° and 64.5° could be indexed to a rhombohedral CuAl-LDH, suggesting the successful synthesis of CuAl-LDH. (JCPDS37-0630; Figure 2d). Some observed peaks corresponding to Cu(OH)_2_ and Al_2_O_3_ were probably due to the decomposition of metal hydroxide at a high temperature. The presence of sharp peaks showed the as-prepared CuAl-LDH had high crystallinity, which was satisfactory because high crystallinity exhibits higher electronic conductivity [38].

### 3.2. Electrochemical Behaviors on CV

**Figure 3:**
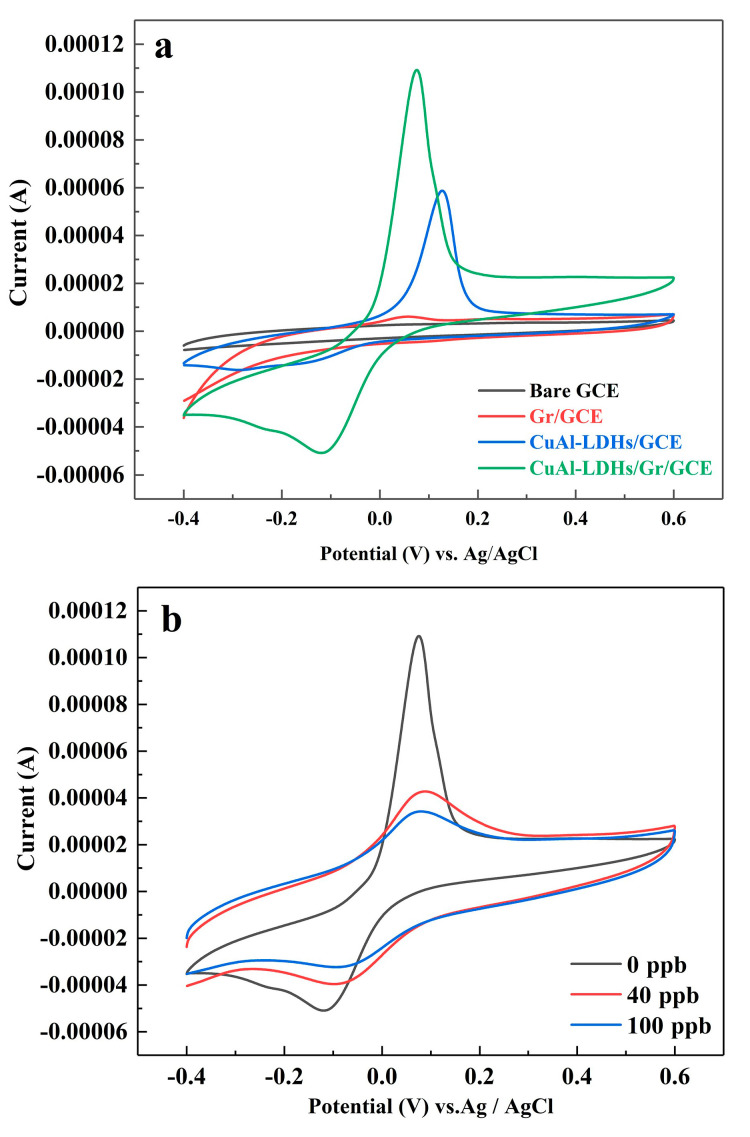
(**a**) Cyclic voltammetry (CV) measurements of the bare electrode, CuAl-LDH, Gr and CuAl-LDH/Gr modified electrodes in BS solution (0.2 M, pH 5.4) and (**b**) CuAl-LDH/Gr/GCE in ABS solution (0.2 M, pH 5.4) with different concentration glyphosate (scan rate 0.05 V/s; sample interval 0.001 V).

The CuAl-LDH/Gr modified electrode had the strongest CV signals when the bare, Gr, CuAl-LDH and CuAl-LDH/Gr modified electrodes was placed in sequence in the 0.1 M KCl solution including 2 mM of [Fe(CN)_6_]^3−/4−^ (Figure S1). This showed that the combination of CuAl-LDH/Gr NCs had the best electronic transmission capability, thus promoting the redox process. Then, the above-mentioned electrodes were immersed in the 0.2 M ABS to record CV responses and no obvious peak was observed in the bare GCE and Gr/GCE (Figure 3a). Carbonaceous material can function easily with covalent or non-covalent bonding metallic nanomaterials to improve electrode sensitivity, but the carbonaceous material alone did not result in a redox peak and electrochemical signals. In the 0.2 M ABS, both CuAl-LDH/GCE and CuAl-LDH/Gr/GCE showed a well-defined oxidation peak from 0.075 to 0.15 V, and the latter peak was more prominent. In previous studies, the oxidized process of Cu(I)/Cu(II) occurred from 0.1 to 0.4 V, therefore the peak here should be ascribed to the stripping process of copper [40,41]. The appearance of the peak verified that the CuAl-LDH worked as the indispensable probe and the addition of graphene effectively enhanced the signals in a related electrochemical reaction.

In following experiments, the stripping peak of the CuAl-LDH/Gr/GCE began to visibly decrease by adding the glyphosate (40 ppb) standard solution to the above ABS solution, (Figure 3b). To further explore this tendency, the glyphosate concentration kept rising to 100 ppb in the ABS. It was observed that CuAl-LDH/Gr/GCE had a lower stripping peak current. It can be inferred that glyphosate molecules inhibit the oxidation of copper. With the higher concentration of glyphosate molecules, the suppressing effect would further aggravate on the CuAl-LDH/Gr/GCE.

### 3.3. Electrochemical Impedance Spectroscopy Tests

**Figure 4:**
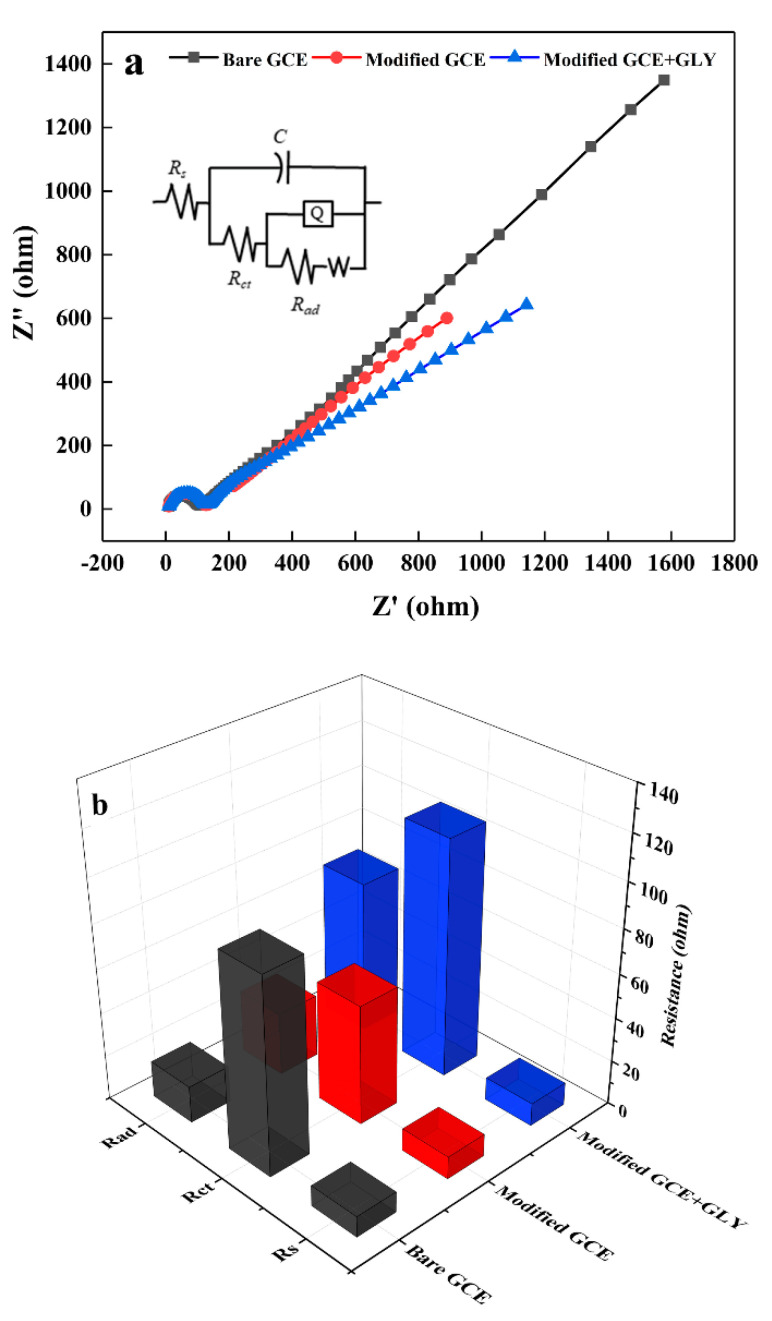
(**a**) Electrochemical impedance spectroscopy (EIS) of the different electrodes in 0.1 M KCl solution (the equivalent circuit was in the inset) and (**b**) the fitting resistance value of the equivalent circuit.

The electrochemical impedance spectroscopy (EIS) was used to study the interaction between glyphosate and CuAl-LDH/Gr NCs. The images of EIS usually include semicircular curves at high frequencies and straight lines at low frequencies [42]. The diameter of the Nyquist semicircular portion at high frequencies reflects the resistance of electron transfer (Ret) [42]. In our study, the Nyquist semicircle of the bare GCE, CuAl-LDH/Gr/GCE and CuAl-LDH/Gr/GCE in the KCl solution with 40 ppb glyphosate increased gradually (Figure 4a). It can be inferred after being exposed to the glyphosate solution, the electrode did change the interfacial property and hinder the electron transfer rate on the surface.

To further explore the mechanism, an equivalent circuit was proposed to match the fitted EIS measurement data for different samples (inset in Figure 4a). Among the equivalent circuit, Rs, Rct and Rad represent the solution resistant, the charge transfer resistance and the adsorption resistance by the glyphosate, respectively; C and Q are related to the interface capacitance and the ideal double-layer capacitance on the electrode surface, respectively and W represents ohmic resistance of Warburg [43]. The EIS experimental data (point in Figure 4a) discussed above were found to be in good agreement with the proposed equivalent circuit (line in Figure 4a) in the Zsimp win program, indicating the equivalent circuit was suitable for the investigated system [44]. The fitting results were shown in Figure 4b. As we used the same electrolyte solution, no significant difference was observed in the solution resistant Rs. The charge transfer resistance Rct increased in the order modified GCE + GLY (108.33 Ω) >  GCE (89.27 Ω) >  modified GCE (54.50 Ω). After coating CuAl-LDH/Gr NCs, the modified electrode denoted the lowest Rct, which gave an indication that the CuAl-LDH/Gr NCs had a positive effect on electrochemical activity and charge diffusion rate. However, after being immersed in glyphosate, some part of CuAl-LDH/Gr deactivated and barrier the charge transfer making the charge transfer resistance Rct doubled. The adsorption resistance Rad had a similar increased tendency. More specifically, the adsorption resistance Rad increased from modified GCE (28.63 Ω) to modified GCE + GLY (69.61 Ω), which was probably because a large amount of glyphosate had been adsorbed on the surface of CuAl-LDH/Gr resulting in even bigger resistance to further adsorption. Therefore, the glyphosate molecules exhibited a strong retarding effect on CuAl-LDH/Gr/GCE, thus leading to the suppressed peak currents, which was consistent with the CV results.

### 3.4. Glyphosate Sensing Mechanism

**Scheme 1:**
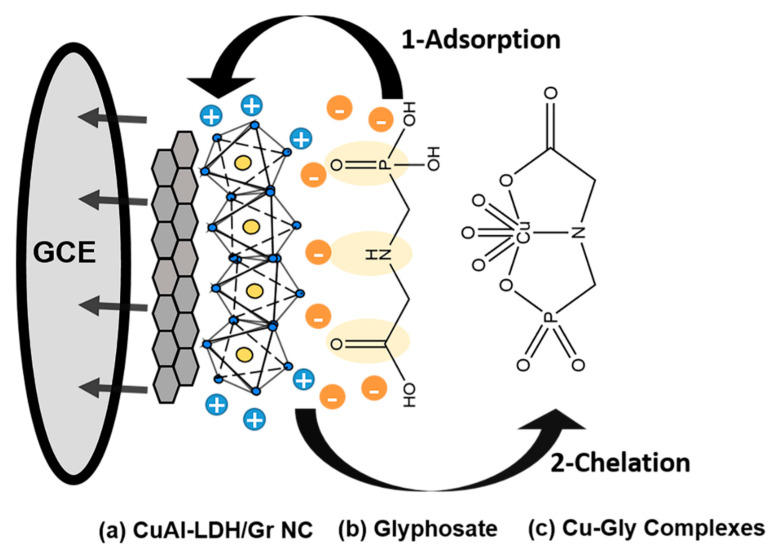
The sensing mechanism of the glyphosate in CuAl-LDH/Gr/GCE.

A reasonable adsorption/chelation sensing mechanism of glyphosate was illustrated in Scheme 1. Initially, CuAl-LDH is driven to reduce the reaction at the deposited potential, then the reoxidation occurs to release the stripping signals by the positive scan. When glyphosate is present in the solution, under the effect of the electrostatic force, the negatively charged glyphosate is adsorbed on the surface of positively charged CuAl-LDH. Further, because specific donor groups (amines, carboxylates and phosphonates) in glyphosate have a strong affinity for copper (II), the adsorbed glyphosate would chelate with copper to form two stable five-membered rings [45]. These Cu-glyphosate complexes have an extremely stable structure, which restrains the copper ions reduction–oxidation reactions and suppresses the stripping signals (the XRD image of Cu-glyphosate complexes shown in Figure S2). In these processes, CuAl-LDH works as an indispensable electrochemical probe and facilitates the high enrichment of glyphosate by a metallic intercalated structure. In addition, as a result of direct mixing of original graphene to preserve its electrochemical property to the greatest extent, the voltammetry responses were effectively enhanced. In general, the synergistic contribution of CuAl-LDH and Gr makes this new combination a promising candidate for evaluating trace levels glyphosate.

### 3.5. Optimization Experiments

#### 3.5.1. Buffer Electrolyte Solution and pH

In order to maintain the concentration of the charged particles, the buffer electrolyte solutions and pH were optimized. Of the two kinds of buffer electrolyte solutions, namely, Na_2_HPO_4_-NaH_2_PO_4_ (PBS) and NaAC-HAC (ABS; pH 5.4), the ABS solution was more suitable because of a more prominent stripping peak, when the same CuAl-LDH/Gr/GCE was used (Figure S3a). This result can be attributed to the less competition of the ion-exchange sites on the electrode surface in ABS solution [46,47]. As glyphosate is easily decomposed and deactivated under alkaline conditions, an acidic pH range of 3.8–5.6 was chosen [48]. The current variance ΔI increased initially and reached its maximum value at a pH of 5.4 (Figure S3b). The pH value not only influenced the surface potential of the CuAl-LDH/Gr NCs but also changed the charge properties of glyphosate. In slightly acidic conditions, the chitosan matrix was positively charged, which was in favor of attracting negatively charged graphene to form a stable film and avoid agglomeration [37]. Furthermore, glyphosate exhibited a global negative charge at pH 5.4, thus enabling stronger electrostatic adsorption to obtain the strongest signals [48]. Hence, the ABS solution (pH 5.4) was considered to provide the best electrolyte condition.

#### 3.5.2. The CuAl-LDH/Gr Ratio and the Volume

In the metal-carbon nanocomposite, graphene was an auxiliary material whose concentration was kept at 150 mg/L to optimize CuAl-LDH concentrations ranging from 63 to 1000 mg/L. Initially, the reduction current ΔI increased significantly with the increasing concentration of CuAl-LDH; however, the reduction value ΔI began to decrease when the concentration increased to more than 500 mg/L (Figure S3c). The suitable concentration of CuAl-LDH could ensure complete voltammetry signals during the preconcentration and stripping process, however excessive metallic material would fade the current amplification effect of Gr inside to transfer electrons due to the steric hindrance. In different volumes of CuAl-LDH/Gr suspension dropped onto the GCE in the range from 8 to 20 μL, the optimal response ΔI was obtained at 16 μL (Figure S3d). The results showed the fact that the excessive volume led to modification overlap, which was unfavorable to the homogeneity of the electrode surface. To make full use of the properties of the metal-carbon nanocomposite, 16 μL CuAl-LDH/Gr suspension with a concentration ratio of 10:3 (500 mg/L:150 mg/L) was considered to be the best choice.

#### 3.5.3. The Deposition Voltage and the Accumulation Time

The deposition voltage and the accumulation time are vital parameters for target glyphosate adsorption. The deposited potential was set from −0.40 to 0.10 V; when the deposition voltage was negative, the peak current reduction ΔI increased accordingly (Figure S3e), which suggested that more glyphosate was attracted to the electrode surface at a more positive deposition potential due to the electrostatic force. However, when the deposition voltage was positive, the deposition voltage was very close to the oxidation potential of copper, which resulted in a decreased peak current and reduced stability. With the initial accumulation time ranging from 30 to 240 s, the peak current dropped rapidly due to further accumulation of glyphosate on CuAl-LDH/Gr NCs (Figure S3f). However, this tendency leveled off slowly after 180 s because of the adsorption saturation of CuAl-LDH/Gr NCs. Considering the accuracy and convenient usage, 180 s accumulation time at −0.1 V deposition voltage was selected for further experiments.

### 3.6. Differential Pulse Voltammetry Detection of Glyphosate

**Figure 5:**
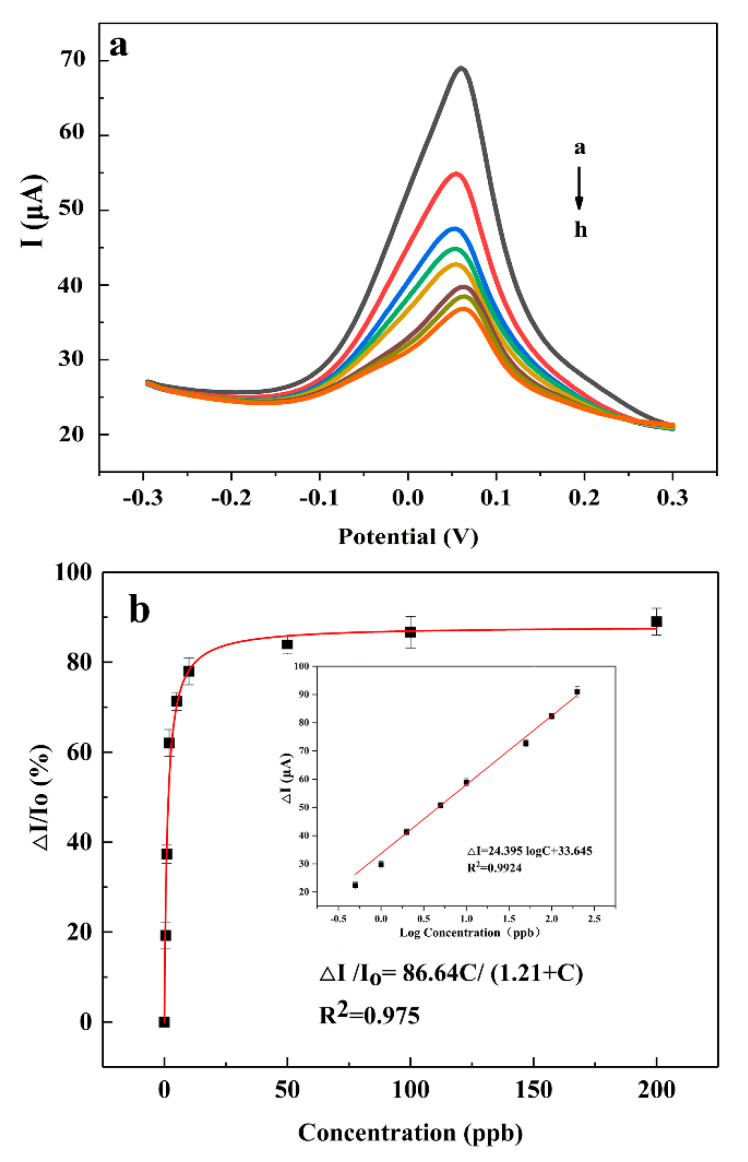
(**a**) Differential pulse voltammetry (DPV) curves of CuAl-LDH/Gr/GCE in 0.2 M pH 5.4 ABS (glyphosate concentrations from a to h, 0.5,1, 2, 5, 10, 50, 100 and 200 ppb; incremental voltage 0.005 V; amplitude 0.05 V; pulse width 0.05 s; sample width 0.025 s and pulse period 0.5 s) and (**b**) the corresponding calibration curve with the Langmuir adsorption fitting curve (the linear curves of the logarithmic value in the above glyphosate concentration and the peak current reduction in the inset).

The electrochemical detection of glyphosate was performed under optimized operating conditions of DPV. The superposition of DPV curves at the increasing concentration of glyphosate was shown in Figure 5a. With the increasing glyphosate concentration, the stripping current declined sharply and the peak potential at +0.495 V showed a slight positive offset. The corresponding calibration curve (ΔI/I_0_ vs. CGly) was obtained based on the stripping signal inhibition percentage (Figure 5b). As the concentration increased, more and more glyphosate was adsorbed onto the electrode surface and the signal inhibition gradually approached saturation. The concentration and the percentages of signal inhibition could be fitted with the Langmuir adsorption formula: ΔI/I_0_ = 86.64C_Gly_/(C_Gly_ + 1.21) (R^2^ = 0.975). According to the Langmuir adsorption theory, the events of glyphosate on the CuAl-LDH/Gr surface were considered as non-interfering single layer adsorption. Further, the logarithmic value of the above glyphosate concentration and the peak current reduction were calculated to fit a calibration equation: ΔI = 24.395logC + 33.645 (R^2^ = 0.9924) (inset in Figure 5b). After calculation, the logarithmic linear range of glyphosate concentration was found to be 0.5–200 ppb (2.96 × 10^−9^–1.18 × 10^−6^ mol L^−1^) and the detection limit was estimated to be 0.169 ppb (1 × 10^−9^ mol L^−1^; S/*n* = 3), which was lower than 6 × 10^−7^ mol L^−1^ in the EU drinking water limit and 0.07 mg/L (4.1 × 10^−7^ mol L^−1^) in the China drinking water limit.

### 3.7. Repeatability, Stability and Anti-Interference

**Figure 6:**
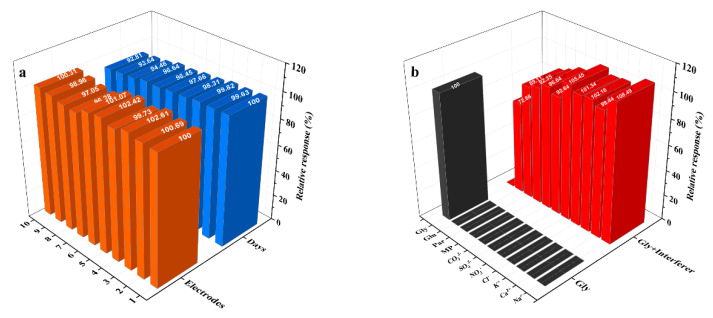
(**a**) Repeatability and stability of CuAl-LDH/Gr/GCE in 0.2 M pH 5.4 ABS solution and (**b**) interference tests of CuAl-LDH/Gr/GCE in 0.2 M pH 5.4 ABS solution with glyphosate and interferers (the concentration of glyphosate was 40 ppb; the other organophosphorus and inorganic ions were separately added to the electrolyte with glyphosate).

The results of the repeatability and stability rests were shown in Figure 6a. The first response of CuAl-LDH/Gr/GCE was regarded as 100% and the relative response was the percentage to the first one. By repeating the experiment ten times in the ABS solution, the sensor demonstrated good repeatability with the relative response ranging from 96.28% to 102.61%. The CuAl-LDH/Gr/GCEs were stored in a dry environment at 4 °C for ten days and the voltammetry responses were investigated every day. After ten-day storage time, the peak current of electrodes maintained 92.83% of initial response with only a slight current decrease in the ABS solution, indicating the good stability of the sensor.

The anti-interference is also the crucial factor for the electrochemical sensor. The response only with glyphosate in the ABS solution was regarded as 100%. The Figure 6b showed the co-existing impact of inorganic ions and organophosphorus pesticides in the ABS solution with 40 ppb glyphosate. From the results, it was found that the impact of inorganic ions (at 100-fold concentration) was insignificant with the response deviation from 92% to 108%.

A slight response increase could be attributed to certain inorganic ions such as Na^+^, promoting the conductive path in the electrolyte. Other organophosphorus (at 10-fold concentration) had a more apparent impact, especially glufosinate, which has a similar chemical structure to glyphosate. The impact of other organophosphorus was acceptable with the deviation below 11.00%.

To verify the applicability of the sensor in actual water samples, the spiked recovery tests were conducted to measure glyphosate. The water samples were collected from Hangzhou city, Zhejiang province, China). The samples were first filtered and centrifuged to remove impurities, and then added acetic acid and sodium acetate to the supernatant to obtain the ABS solution (0.2 M, pH 5.4). Thereafter, three different spiked concentrations of glyphosate with three parallel experiments were detected by DPV. The recoveries ranged from 97.64% to 108.08% and deviations were calculated as less than 5% (Table 1), which revealed the practicality and accuracy of the proposed sensor.

We compared our sensor with other glyphosate detection methods (Table 2). It was found that our sensor had a relatively wider detection range and significantly lower detection limit than any other mass spectrometers especially the spectrophotometer [16,21,49]. Compared to AuNPs and enzymes modifications, the CuAl-LDH/Gr NCs had the advantage of low cost and simple preparation process, but retained the excellent sensitivity and conductivity [16,18,50]. In addition, our sensor can meet the requirements of long storage time and complex environmental conditions.

## 4. Conclusions

A novel non-enzymatic electrochemical sensor for trace glyphosate was successfully fabricated using a CuAl-LDH/Gr NCs modified electrode. The as-prepared CuAl-LDH/Gr NCs makes use of CuAl-LDH as a sensitive probe combining the advantage of graphene (high conductivity and large surface area). The laboratory data showed that the sensor had a wide logarithmic linear detection range with high repeatability, stability and good anti-interference for ions and organophosphorus pesticides. In the real water, the results with the recovery percentage of 97.64%–108.08% verified the sensor accuracy and practicality. The electrochemical sensor expands the application of LDH and provides a feasible method for glyphosate determination in situ.

## Figures and Tables

**Table 1:** Recovery studies in actual water samples.

Sample	Detected (ppb)	Spike (ppb)	Found (ppb)	Recovery (%)	RSD (%)
1	0	40	41.66	104.15	4.15
2	0		39.78	99.45	
3	0		43.23	108.08	
4	0	80	78.62	98.28	3.21
5	0		79.26	99.08	
6	0		81.37	104.21	
7	0	120	125.30	104.42	4.28
8	0		117.17	97.64	
9	0		127.04	105.87	

**Table 2:** Comparison of the sensor with other glyphosate detection works.

Method	Materials	LOD (mol L^−1^)	Linear Range (mol L^−1^)	Ref
Fluorescence	Co-B/SiO_2_/dye NPs	2.5 × 10^−10^	1.0 × 10^−9^–1.0 × 10^−2^	[51]
Colorimetric	PVNA ^1^	5.9 × 10^−7^	5.9 × 10^−6^–2.9 × 10^−3^	[49]
Potential biosensor	A-G@urease/AuNPs ^2^	5 × 10^−7^	5 × 10^−7^–5 × 10^−5^	[16]
Amperometry biosensor	CNO/Tyr ^3^	6.5 × 10^−9^	1.5 × 10^−9^–1.0 × 10^−5^	[18]
Electrochemical voltammetry	AuNPs/MIP ^4^	2.1 × 10^−9^	2.4 × 10^−8^–1.0 × 10^−5^	[50]
Electrochemical voltammetry	NiAl-LDH ^5^	1.0 × 10^−6^	1.0 × 10^−5^–9.0 × 10^−2^	[21]
Electrochemical voltammetry	CuAl-LDH/Gr NCs	1.0 × 10^−9^	2.96 × 10^−9^–1.18 × 10^−6^	This work

^1^ the poly (vinyl) alcohol (cd-PVA (copper doped poly (vinyl) alcohol)) nanofiber; ^2^ agarose-guar gum entrapped bio-nanoconjugate of urease with gold nanoparticles; ^3^ carbon nano-onion/tyrosinase; ^4^ gold nanoparticles/molecularly imprinted polymers; ^5^ Ni_1−x_Al_x_(OH)_2_NO_3x_·nH_2_O layered double hydroxide thin film.

## Supplementary Materials

The following are available online at https://www.mdpi.com/1424-8220/20/15/4146/s1, Figure S1: Cyclic voltammetry curves of 2 mmol L^−1^ [Fe(CN)_6_]^3−^/^4−^ in 0.1 mol L^−1^ KCl solution; Figure S2: The XRD image of Cu-glyphosate complexes; Figure S3: Optimization experiments with different influencing factors in DPV.
