# Supplementary material for: Performance of CuAl-LDH/Gr Nanocomposite-Based Electrochemical Sensor with Regard to Trace Glyphosate Detection in Water
Source: Sensors (Basel). 2020 Jul 25;20(15):4146. doi: 10.3390/s20154146 (PMC7435834; doi:10.3390/s20154146)
Supplement: Supplementary file 1 [file sensors-20-04146-s001.docx]

Supplementary Material: 3 Figures.

Performance of CuAl-LDH/Gr nanocomposite-based electrochemical sensor with regard to trace glyphosate detection in water

Chuxuan Zhang ^1^, Xinqiang Liang ^1,2^* and Yuanyuan Lu ^3^ and Xiangyang Xu ^1,2^

^1^ College of Environmental and Resource Sciences, Zhejiang University, Hangzhou 310058, China;

^2^ Key Laboratory of Water Pollution Control and Environmental Security Technology, Zhejiang Province, Hangzhou 310058, China;

^3^ Physical and Theoretical Chemistry Laboratory South Parks Road University of Oxford, OX1 3QZ, England, United Kingdom;

***** Correspondence: liang410@zju.edu.cn; Tel.:86-0571-88982809





**Figure S1** Cyclic voltammetry curves of 2 mmol L-1 [Fe(CN)_6_]^3-/4-^ in 0.1 mol L-1 KCl solution. (CV tests: scan rate 0.05 V/s; sample interval 0.001 V; potential range -0.4- 0.6 V)





**Figure S2** The XRD image of Cu-glyphosate complexes


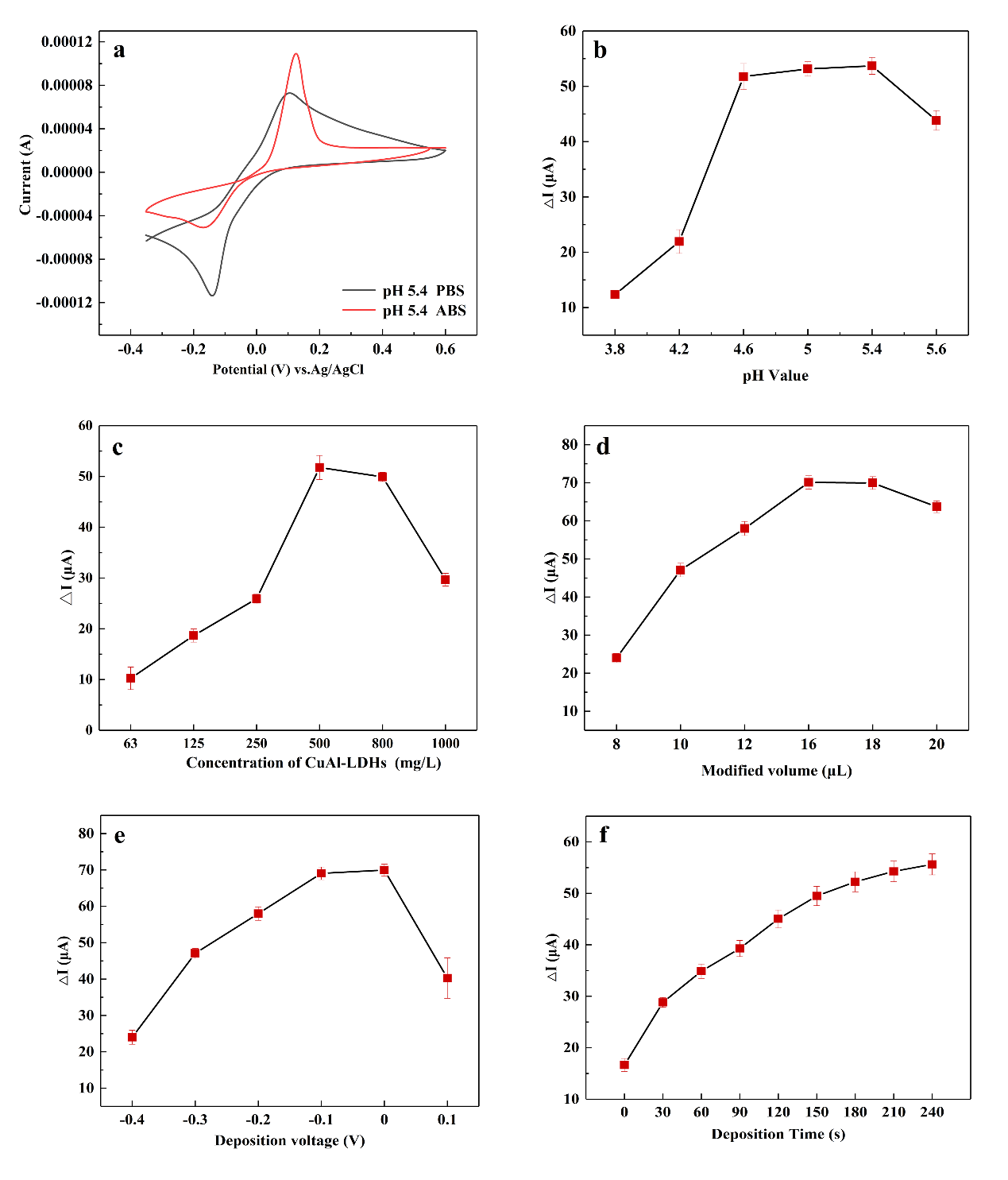


**Figure S3** Optimization experiments with different influencing factors in DPV: the electrolyte solution (a); the pH (b); the CuAl-LDHs /Gr ratio concentration (c); the modification volume (d); the deposited voltage (e); the accumulation time (f).
